# Looking for patterns of change amid pandemic period in students’ evaluation of academic teaching

**DOI:** 10.1007/s11135-022-01567-7

**Published:** 2022-11-18

**Authors:** Annalina Sarra, Adelia Evangelista, Barbara Iannone, Tonio Di Battista

**Affiliations:** Department of Philosophical, Pedagogical and Economic-Quantitative Sciences, University “G.d’Annunzio”, Viale Pindaro, 42, Pescara, 65122 Italy

**Keywords:** IRT, Linear logistic models with relaxed assumptions, Higher education, Online learning, Learning satisfaction data

## Abstract

The COVID-19 pandemic has shaken not only the global economy but every development field, including all levels of the education sector and in every place in the world. The wide spread of this pandemic disaster has undoubtedly changed the education landscape worldwide. Online teaching and learning become the primary instruction method and the global world of schools, colleges and universities were forced to adapt this model. The first concern about online learning is whether this method is effective compared to traditional face-to-face lessons. In this paper, we carried out a quantitative analysis to explore variations in university students’ feedback on learning experience in the context of this new challenging situation caused by the COVID-19 pandemic. By adopting an IRT modeling, we compared the appreciation of some aspects of 41 courses taught at the University of Chieti-Pescara (Italy) during the educational emergency with that of the previous year. Overall, from the results of this study, it is arisen that students have given positive feedbacks on their learning experiences and their effectiveness.

## Introduction

The coronavirus disease (COVID-19) spread rapidly worldwide and it was declared, by the World Health Organization, as a pandemic phenomenon on March 11, 2020. The COVID-19 pandemic brought a global crisis, and its consequences to date have been severe and significant on all sectors of our lives, especially on health and economy, causing, among others, acute economic loses and a critical pressure on health systems. Response to the pandemic has been widely different from country to country, depending on the alignment to the national government and their pre-existing investment in resilience. In the educational context, the immediate impact of COVID-19 has been lockdown and the enforced closure of educational institutions. Schools, colleges and universities have been shut down across the world and forced to implement a transition from traditional face-to-face teaching methods to online teaching or a combination of online and traditional teaching (blending), to ensure continuity of education (Aristovnik et al. 2020; Flores et al. 2020, 2021; Watermeyer et al. 2021). During the COVID-19 pandemic, e-learning, as a distance learning strategy, enabled universities to use a variety of online learning platforms; Zoom, Google classroom, Microsoft Teams, Blackboard are some of the most adopted versatile platforms for online learning and teaching processes during the ongoing outbreak. According to recent reports, COVID-19 has caused an education crisis (Hodges et al. 2020). Different published works have already documented that the emergency of online learning has resulted in many difficulties for teachers, students and higher education administrators (Chung et al. 2020; Korkmaz and Toraman 2020; Xiong et al. 2020). While some nations, thanks to their economic and human resources, organizational skills and students’ ability to interact with the electronic environment, have been able to easily shift to online learning, for others the transition from face-to-face to online teaching was fraught with difficulties. The limited availability to computers and internet resources, and thus some problems in accessing online lessons, materials downloading, the lack of digital skills in using online platforms, are the main challenges and obstacles encountered by the students. Other studies have tried to examine the feedbacks of universities to this new challenge all over the world (Guangul et al. 2020; Harley et al. 2021; Metcalfe 2021).

For instance, a research carried out by Crawford et al. (2020) outlined the reaction of several universities through countries and demonstrated diverse responses to the complex challenge. Overall, the ability of colleges and universities to quickly shift toward online and virtual courses is impressive. At the same time, the effects on teaching and learning has been mixed (Dhawan 2020; Liguori and Winkler 2020; Shahzad et al. 2021; Tasso et al. 2021; Vicente et al. 2020; Wang et al. 2020).

Additionally, further comparative surveys were aimed at exploring whether face-to-face or traditional teaching methods are more productive or whether online or hybrid learning is better (Gonzalez-Gomez et al. 2016; Lockman and Schirmer 2020; Pei and Wu 2019). Findings arising from these studies give evidence of a better students’ performance in online learning than in traditional one. Besides, in the past, several researches have been carried out to explore students’ satisfaction with online learning teaching, which was used as a pedagogical method for many years in various fields (Bolliger and Halupa 2018; Martin and Bolliger 2018; Palvia et al. 2018; Pereira et al. 2017; Selwyn 2014; Stevens and Switzer 2006).

In this regard, it is worth noting that the both proponents and opponents of online learning continued, over time, the discourse surrounding quality of online learning provisions, student's engagement and satisfaction (Carini et al. 2006; Fredricks et al. 2016; Gordon et al. 2015; Gray and DiLorento 2016).

However, a limited literature is available on students’ satisfaction with online experiences during the coronavirus pandemic (Rajabalee and Santally 2020; She et al. 2021; Szopiń ski and Bachnik 2022; Zheng et al. 2021).

To fill this gap, in this paper, we explore variations in students’ feedback on learning experience in the context of this new challenging situation of digital learning caused by COVID-19 disease. Using quantitative data obtained through the administration of the same questionnaire to face-to-face and online learners, this research compares the appreciation of the courses of University of Chieti-Pescara (Italy) during the educational emergency with that of previous year. Specifically, the approach followed in the present research seeks to investigate three hypotheses:

- **Hypothesis 1 (H**$_1$**)** Teaching aspects positively affecting students’ satisfaction. We were aimed to answer the following question: “What are the teaching aspects that play a pivotal role in students’ satisfaction?”
- **Hypothesis 2 (H**$_2$**)** Teaching aspects undergoing an improvement or a worsening during the COVID-19 impact on education. We are aimed to answer the following question: “How did online learning during the second semester (May 2020) impact students’ satisfaction, as compared with a previous analogous cohort who received face-to face instruction in the first semester (December 2019)?”
- **Hypothesis 3 (H**$_3$**)** Quantifying the dependence of temporal changes on some covariates effect. We were aimed to answer the following question: “Are the temporal changes affected by the grouping of teaching courses in humanistic and scientific classes?”

We address these research questions through the Item Response Theory (IRT) modelling techniques. IRT (De Mars 2010), successfully applied in many settings, refers to a family of probabilistic models that attempt to explain the relationship between latent traits (e.g. attitudes, abilities, skills, satisfaction) and their manifestations. IRT assumes that the latent construct and the items of a measure are organized in an unobservable continuum. In our context, we exploited the attractive features of a particular class of IRT models, known as Linear Logistic Models with Relaxed Assumptions (Fischer 1976), to detect patterns of change amid pandemic period in students’ evaluation of academic teaching compared to the previous year. The remainder of this paper is organized as follows. Section 2 is devoted to illustrate the challenges that universities are facing with over the last years, and the importance of the assessment of quality within higher institutions. Section 3 covers the basics of IRT models adopted in this study to capture the change amid pandemic period in students’ evaluation of academic teaching. Data and main results are then presented in Sects. 4 and 5, respectively. Finally, Sect. 6 concludes the paper.

## Background: quality within higher education institutions

Universities, both nationally and internationally, have experienced a profound evolution over time, often under the pressure of reforms desired by individual States (Aarrevaara et al. 2009; Boitier and Riviere 2013; Engwall 2007; Krejsler 2006; Naude and Ivy 1999). In particular, in the last years, many universities are in front of important challenges, regarding the decline of enrolments, an increased competition, the low number of graduates in the labour force, high rate of dropout, other than drastic financial troubles. Furthermore, in the new context, the funding State calls for quality services by setting specific targets. Various indicators are in fact mirrored in funding formulae and public funding is delivered to universities according to different parameters (Patterson 2001). The ways higher institutions are organized, financed and delivered are at the basis of their complexity. Universities are open systems that establish relationships with the environment. Such relationships, in terms of constraints and opportunities, must be considered as a strategy to creating public value (Mussari et al. 2015; Rebora and Turri 2009; Deidda Gagliardo 2015). Over the past decades, quality of teaching and learning has become a major strategic issue in tertiary education systems across the globe (Donna 2018). Universities are increasingly aware of the importance of students’ satisfaction for reputation as well as for educational reasons (Palihawadana 1999). The perceived quality of the educational experience is primarily important as diagnostic feedback to administrators and faculty for improving teaching; in addition, it provides information for students in the selection of courses and instructors. Mapping the quality of teaching is necessary, at least in principle, also for the government bodies to modulate the allocation of resources for higher education institutions, according to pre-assigned parameters. In a word, in a very competitive global educational marketplace, having satisfied “customers” has become an important component of quality assurance and quality enhancement (Rienties 2014). During the last decades, in the Italian context, there has been both an increased pressure on service quality (Paletta 2005; Rebora 2003) and a rising interest in evaluating university courses (Pagani and Seghieri 2002; Colombu et al. 2021; Bertaccini et al. 2021). Since 1999, each Italian public university has begun to monitor and assess the quality of its own teaching activities, with the overall aim of improving the quality of services offered to students (Paolini and Del Bene 2021).

The fundamental regulatory hub that gave rise to the latest changes can undoubtedly be identified in Law no. 240/2010, known as the Gelmini Reform Dal Molin et al. (2017). In order to increase the quality and efficiency of the university system, this rule has profoundly changed the governance structures, funding mechanisms and staff recruitment of the universities. Likewise, with a view to periodic evaluation of the study courses themselves, the ministerial legislation provides for a large set of indicators. In the new set of indicators outlined by Law 240/2010, we can distinguish those related to teaching, internationalization and evaluation of teaching. A continuous and systematic check of the university’s performance in the organization, research and teaching activities is also carried by the Evaluation Unit with the overall goal of the improvement of the internal self-assessment and the promotion of merit. Besides, it should be considered the periodic accreditation, on a three year basis, of study courses. The accreditation decisions are taken consistently with a set of additional requirements, that constitute ANNEX 8 of the AVA 2.2 guidelines, currently in force (www.anvur.it). For these reasons, the assessment of quality within higher education institutions have gained in frequency and proceeds apace. Universities systematically collect learning satisfaction data and measure students’ satisfaction in order to monitor and improve the teaching and learning experience (Arbaugh 2014) and to identify their strengths and areas for enhancement (Eom et al. 2006; Marsh 1982; Zerihun et al. 2012). Student's evaluation of teaching is an acknowledged instrument, used to map the quality of teaching in universities and advance the chances that desired learning outcomes will be attained (Emerson et al. 2000; Kember and Ginns 2012; Onwuegbuzie et al. 2007).

One of the widely used methods for evaluating courses, programmes and teaching is represented by students’ ratings, that are commonplace in many universities. Students’ opinions, whose validity has been sufficiently well established (see, among others, Arubayi 1987; Kulik 2011; Marsh 1984), are a vital source of information for both universities and government bodies. Collecting students’ evaluations on the perceived quality of teaching necessarily requires to take into account the latent construct of the variable of interest. Students’ ratings, provided on a set of items and typically expressed on an ordinal scale, are in fact indirect measurement of the quality of the university courses. Thus, instead of assigning them arbitrary scores and analyzing them through linear models, it is desirable to recognize their discrete nature and provide data analysis via probabilistic models (Rampichini et al. 2004), such as those of the Item Response Theory (IRT) class. IRT provides a convenient and versatile family of measurement models concerned with the measurement of an individual’s latent trait, such as attitude, ability, skill, satisfaction with a product or service, assessed indirectly by a group of items (De Mars 2010; Reckase 2009). Their mathematical characteristics allow a transformation from binary or ordinal answer pattern, e.g. Likert type data, into a measure on an equal-interval scale (de Ayala 2009). Following this line of research, the focus of the study is to look for patterns of change amid pandemic period in students’ evaluation of academic teaching. To this end, a particular class of IRT models, described in the next section, assumes relevance.

## Methodological framework: IRT with relaxed assumptions

IRT encompasses a variety of statistical methods, differing each other in terms of item characteristics that are included in the model and in terms of the response option format (De Mars 2010).

All IRT models include a latent trait parameter and one or more item location parameters, and link the probability of a subject *i* ($i=1, \ldots ,N$) of providing a certain answer $Y_{ij}$ to an item *j* ($j=1, \ldots ,J$), given the individual latent trait and one or more item characteristics.

The basic IRT model for dichotomous data (and a single dimension), also known as the one-parameter Logistic Model (De Boeck and Wilson 2004), or Rasch Model, can be expressed as:

1 $$\begin{aligned} Pr(Y_{ij}=1|\theta _{i},\beta _{j})=\frac{exp(\theta _{i}-\beta _{j})}{1+exp(\theta _{i}-\beta _{j})} \end{aligned}$$

In the Rasch Model, we have *J* parameters $\beta _{j}$, one for each item, and *N* parameters $\theta _{i}$, one for each subject. In the original application, that is the educational measurement, the $\beta _{j}$ parameters are a linear continuous measure of “difficulty” and the $\theta _{i}$ parameters are a linear continuous measure of “ability”. The essence of Rasch modelling is to bring the difficulty of item and the ability of subject to the same logit scale. It is worth noting that the meaning of item and person parameters needs to be specified in accordance with the latent trait considered.

In our context, borrowing the interpretation from the literature on service quality and customer satisfaction, the item parameter represents the “intrinsic quality” of each particular teaching aspect considered, while the person parameter has an interpretation in terms of satisfaction. An IRT model for dichotomous data operates under the assumption that the items share a common underlying construct (*unidimensionality*), i.e. it is supposed that the item responses are explained by one latent trait, and *local independence*. This last condition implies that, given the value of the latent trait, the item responses are distributed independently.

The dimensionality assumption can be checked by means of the generalized Martin-Löf test (Glas and Verhelst 1995), that is a likelihood test for comparing the null hypothesis of unidimensionality against the alternative hypothesis of two dimensions. Furthermore, to gauge whether individual items fit the Rasch model, fit statistics are widely used (Smith and Suh 2003). The two commonly used mean square fit statistics are the Infit mean square (also referred to as the weighted mean square) and the Outfit (or unweighted) mean square. The Infit and Outfit mean squares can be converted to an approximately normalised t-statistic. These Infit/Outfit t-statistics have an expected value of 0 and a standard deviation of 1 and the results between + 2 and $-$ 2 suggest that observed data follow the Rasch model while values greater than + 2 are interpreted as demonstrating more variation than predicted.

Motivated by a broad range of applications across different scientific disciplines, considerable advances have been made in the IRT research over the last decades. Particular relevant for the present paper, is the work conducted to using IRT models for measuring change over time. When the objective is to monitor the trend of a latent variable to understand if significant change occurs, different models, derived from the Rasch models, can be used. Specifically, the Linear Logistic Test Model (LLTM), the Linear Logistic Models with Relaxed Assumptions with the hybrid version (HLLRA) and Mixed Rasch Models (MRM) (Fischer 1973, 1983; Scheiblechner 1972), have been proposed for almost three decades now for the analysis of repeated measure designs, even if not all of them were primarily developed for measuring change.

Here, we restrict our attention to the Linear Logistic models with Relaxed Assumption (LLRA), firstly introduced by Fischer (1976), whose underlying assumptions are less restrictive than for the other models. LLRA models, in fact, require neither unidimensionality of items nor distributional assumption about the latent trait. Besides, LLRA models provide a flexible framework for modeling change as a function of trend effects, covariate/treatment main and interaction effects (Hatzinger and Rusch 2009). This gives the benefit to test specific hypotheses, for example weather there is a trend effect or weather an experimental group shows more improvement than the control one. Typically, in applications dealing with the measurement of change, the same “real” items are presented several times.

For sake of simplicity, we focus on a model for dichotomous response at two time points $T_{1}$ and $T_{2}$. A very straightforward paradigm for measuring change is to describe any change of latent variable as a change of item parameters. More in detail, LLRA formalize the above-mentioned effects by a linear decomposition of “virtual items”. An item $I_{j}$ presented on two occasions to the same person can be conceived as a pair of “virtual items” $I_{a}^{*}$ and $I_{b}^{*}$, with associated “virtual” difficulty parameters $\beta _{a}^{*}$ and $\beta _{b}^{*}$. By assuming that the amount of change between $T_{1}$ and $T_{2}$ is equal to a constant $\delta$, over all subjects, it results that the item parameters are a linear combination of the real item parameter and the change effect. Hence, the pair of “virtual items” generated by the real item $I_{j}$ with difficulty $\beta _{j}$ is characterized by the two parameters $\beta _{a}^{*}=\beta _{j}$ and $\beta _{b}^{*}=\beta _{j}+\delta$. Let us denote with $i=1, \ldots ,N$ the subjects, *t* the measurement occasion and $\theta _{it}$ the “ability parameter” for *i*th person at time *t*. A LLRA model is described by the following equations:

2 $$\begin{aligned}&\Pr (Y_{ij1}=1|T_{1},\theta _{ij},\beta _{j})=\frac{exp(\theta _{ij}-\beta _{j})}{1+exp(\theta _{ij}-\beta _{j})} \end{aligned}$$

3 $$\begin{aligned}&\Pr (Y_{ij2}=1|T_{2},\theta _{ij}^{*},\beta _{j})=\frac{exp(\theta _{ij}^{*}-\beta _{j})}{1+exp(\theta _{ij}^{*}-\beta _{j})}=\frac{exp(\theta _{ij}+\delta _{ij}-\beta _{j})}{1+exp(\theta _{ij}+\delta _{ij}-\beta _{j})} \end{aligned}$$

where $Pr(Y_{ij1}=1|T_{1},\theta _{ij},\beta _{j})$ and $Pr(Y_{ij2}=1|T_{2},\theta _{ij}^{*},\beta _{j})$ are the probabilities for subject *i* to score 1 on item *j* at $T_{1}$ and $T_{2}$ respectively, while $\theta _{ij}$ (the latent trait) is the location of subject *i* on *j*th item at $T_{1}$ and $\delta _{ij}=\theta _{ij}^{*}-\theta _{ij}$ is the quantity of change, on item *j* for *i*th subject.

The flexibility of LLRA arises from a linear reparametrization of $\delta _{ij}$ to include different effects. Following Hatzinger and Rusch (2009), the quantity $\delta _{ij}$, that is the sum of all changes that subject *i* undergoes between two time points, can be modelled as the sum of group-specific (treatment) effect parameters and an unspecified trend parameter, i.e. changes independent of the treatment, e.g. retest effects. The re-parametrization equation is

4 $$\begin{aligned} \delta _{ij}=\sum _{s}q_{isj}\lambda _{sj}+\tau _{j}+\sum _{s<l}q_{isj}q_{ilj}\rho _{slj} \end{aligned}$$

In Eq. (4), $q_{isj}$ stands for the dosage of treatments *s* for item *j* in subject *i*, $\lambda _{sj}$ denotes the effect of the treatment *s* on item *j*, $\tau _{j}$ is the parameter for the trend effect on item *j*, between $T_{1}$ and $T_{2}$, and $\rho _{slj}$ are the parameters for interaction effects of treatments *s* and *l* on item *j* (Hatzinger and Rusch 2009). The interested reader is referred to Fischer (1974) and Fischer (1995), where a more accurate description of LLRA models is provided. Here, it is sufficient to say that the parameter estimation in a LLRA model is based on conditional maximum likelihood estimation (Mair and Hatzinger 2007b).

## Data

As previously pointed out, this study is aimed at assessing variations in students’ feedback on learning experience after the unplanned and rapid interruption of face-to-face education due to COVID-19 pandemic. In response to the COVID 19 pandemic, courses moved to remote instruction on 11st March 2020. The research was conducted at the University “G.d’Annunzio” of Chieti-Pescara (Italy). Overall, 41 teaching courses, activated at the Department of Philosophical, Pedagogical and Economic-Quantitative Sciences, have been evaluated. Most of the courses belong to the humanistic area (60%), whereas the 40% pertain to the scientific subjects. The number of enrolled students at the 41 courses is 2403. From the university register, we elicited some students characteristics: gender, age, level of study and area of residence. Demographic mix of students population is reported in Table 1. It results that the gender of 70% of students are female and 30% are male. Table 1 shows that the age of more than 50% of them are between 19 and 24 years and about 13% of them are above 35 years old. Almost the number of enrolled students from all study levels are equal. Most of the students (60%) are from Abruzzo and the rest from other regions. Additionally, administrative records contain information about the high schools that students attended throughout their educational careers. We found out that approximately 40% of them attended five years of lyceum while 50% come from technical and professional high schools. Students’ satisfaction with some features of university teaching, was assessed quantitatively using an online survey. Face-to-face and online learners of the same courses, taught in two subsequent semesters, before and during the lockdown, were encouraged to fill the questionnaire at the end of regular class times to enhance the learning experience. Specifically, the online survey was administered on two different measurement occasions: at the end of first term (December 2019) and at the end of second term (May 2020). Respondents were informed that survey is anonymous and their responses would be reported in aggregated form for research purposes. Out of 2403 of enrolled students, 1606 have answered the questionnaire. The survey included questions mainly related to the satisfaction with the course topic, the teaching load, the adequacy of learning material and its content, the respect on schedule with program, the clearness of exams rules, the teacher’s clarity and teacher’s ability to prompt student’s interest in the lecture and the teacher’s availability during the lesson and/or reception to provide clarifications on the topics covered.

**Table 1:** Demographic mix of enrolled students

	N.	%
*Gender*		
Male	731	30
Female	1672	70
*Age*		
19–22 ys	743	30.9
22–24 ys	503	20.9
24–27 ys	449	18.7
27–35 ys	401	16.7
Over 35 ys	307	12.8
*Level of study*		
Level 1	822	34.2
Level 2	829	34.5
Level 3	752	31.3
*Residence*		
Abruzzo	1480	60
Other regions	923	40
*High School Degree*		
Lyceum	950	39.5
Technical Schools	512	21.3
Professional Schools	145	6
Other	796	33.1

The items were measured on four categories according to the Likert scale: definitely no (1), more no than yes (2), more yes than no (3), definitely yes (4). The English version of the questions administered to the students are provided in the “Appendix”. The questionnaire on the perceived quality of teaching does not allow to gather students’ background data. Accordingly, without losing in generality, we can infer representative socio-demographic information of respondents by applying population weights to the survey sample. It is important to stress that the IRT modelling, reviewed in Sect. 3, and able to track latent trait change over time, is applicable in measurement contexts in which items are administrated more than once to the same individuals. In our application, given that it is not possible to rely on the same group of students to acquire opinions about university teaching satisfaction over the two considered measurement occasions (Spring 2020 semester and the semester before), we refer to each teaching course as “subject” in the IRT framework jargon. The starting point for carrying out the analysis was the computation of the overall median of item scores at two time points. To create binary data, responses to the items have been dichotomized according to the overall median value.

## Results

In this section, we summarize the main results obtained in assessing the impact of COVID-19 pandemic in university teaching satisfaction. Quantitative data gathered from the online survey were analyzed using *eRm* R package (Mair and Hatzinger 2007a).

Before assessing the teaching course satisfaction during the transition from traditional face-to-face to online learning, we measure the teaching quality carrying out the dichotomous Rasch model analysis. Here, teaching quality is viewed as a unidimensional latent construct, investigated through seven items, listed in Table 2.

**Fig. 1:**
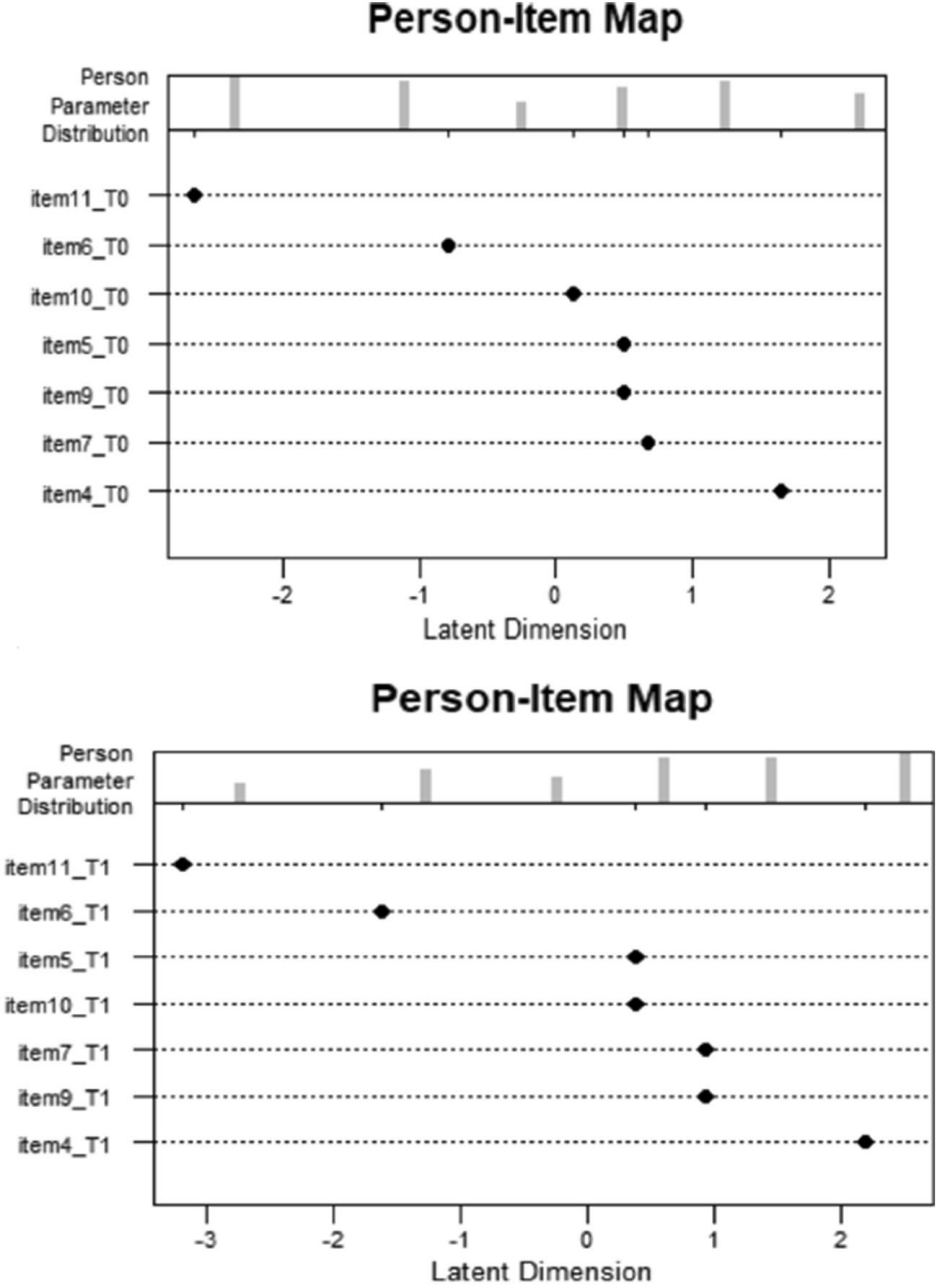
Item-person map at time $T_{0}$ (top panel) and $T_{1}$ (bottom panel)

Firstly, for a meaningful interpretation of dichotomous Rasch model estimates, we check whether data approximate the key requirements for the model. An inspection of Martin-Löf test results suggests that the assumption of unidimensionality is tenable on both temporal occasions. Next, to investigate how well data conformed to the Rasch model and identify mis-fitting items, we also consider Rasch fit statistics . In Tables 3 and 4, we display the Infit mean square (also referred to as the weighted mean square) and the Outfit (or unweighted) mean square. As we can see from Table 3, item 4 at time $T_0$, is outside the acceptable range of Outfit and Infit statistics. This result indicates that individuals may be responding different than the model predicts and implies a lack of unidimensionality that should be investigated. A graphical representation, useful to discovery how persons’ distribution relates to the items, is given by the Person-Item map. In an ideal situation, this map should display item parameter and person parameter mirroring each other about a mean of 0 logits. For our data, the Person-Item maps are shown in Fig. 1 and they highlight that there are areas of latent construct poorly assessed by the items.

**Fig. 2:**
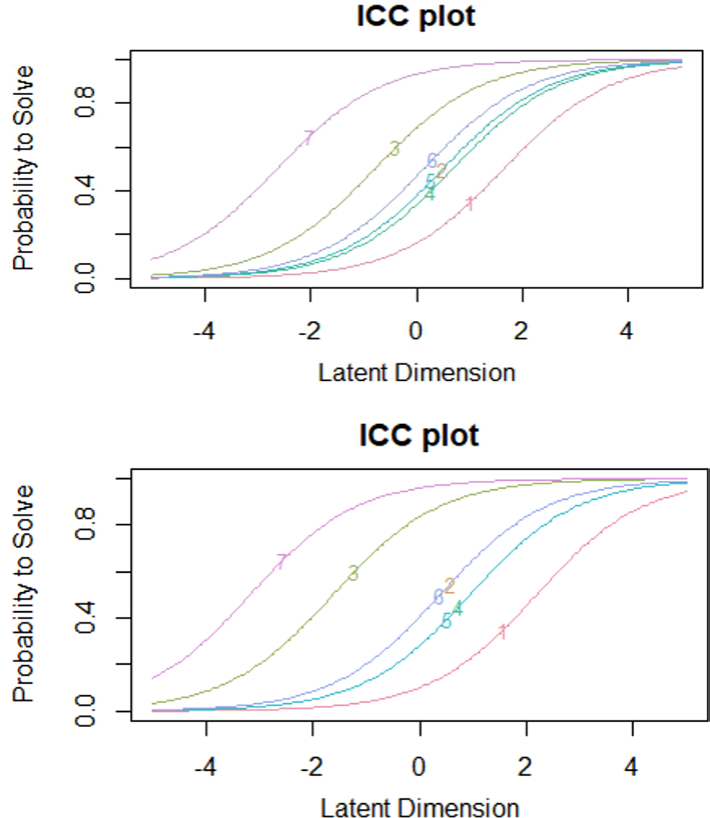
Item characteristic curves at time $T_{0}$ (top panel) and $T_{1}$ (bottom panel)

Our first hypothesis concerned the investigation of items that mostly influenced the students’ learning satisfaction. The step in the Rasch analysis looking at the item parameter estimates allows to check $H_1$. The results of item parameter estimates at time point $T_0$ and $T_1$ are shown in Table 5. It is important to recall that the smaller values of these estimates describe the “easiest items”, that is features of the teaching with a satisfactory quality level. At both times, $T_0$ and $T_1$, we see that the items to which are associated the highest level of quality are those investigating teacher’s availability in providing clarification on the topics (item 7, $\beta _{T0}=-2.639$ and $\beta _{T1}=-3.182$ ) and teacher’s respect or schedule of lessons, exercises, didactic activities (item 3, $\beta _{T0}=-0.793$ and $\beta _{T1}=-1.628$). The Item Characteristics Curves, displayed in Fig. 2, allow to compare and identify features with satisfactory and unsatisfactory quality level.

The second hypothesis supposed that there are items that can exhibit a positive or negative value in terms of temporal trend, during the transition from face-to-face instruction to online learning. In ascertaining if teaching facets undergone an improvement or a worsening when there was the shift to online distant learning, we have to look at the Fisher’s LLRA procedure estimates. To continue this investigation, we need to properly defining the design matrix (Hatzinger and Rusch 2009). Initially, to inspect for change over time, we specify a model where the trend is supposed to be different for all items. In our context, we estimate seven change parameters $\tau$. Results summarised in Table 6, suggest that between the two time points all items experienced an improvement in terms of intrinsic quality. In fact, we record significantly positive values for all trend parameters. We also specify a model whose underlying hypothesis is to assume only one trend parameter for all items. For the model, that generalises the trend over all items, the resulting estimate of $\tau$ is equal to 1.455 with SE = 0.242. The two competing models have been compared by performing a likelihood ratio test. The result of test (Likelihood ratio statistic: 7.16, df = 6 *p* = 0.305) supports the model with just one change parameter for the items.

The third hypothesis concerned the assessment of the dependence of temporal changes on some covariates effect. To test $H_3$, our analysis of the effects of COVID-19 pandemic lockdown on academic teaching satisfaction is completed through the specification of a further model where, in addition to the temporal effects, we estimate the influence of certain covariates over time. Specifically, our aim is to assess to what extent the temporal changes may be affected by the grouping of teaching courses in humanistic and scientific classes. To this end, we adopt a LLRA model with a simplification of the re-parametrization of quantity of change. It follows that Eq. (4) only includes time (trend) and covariate effects. We assume as “control group” the humanistic class, while the scientific class is the “treatment group”. For this latter model, we found significant estimates for item 1 and item 4. Focusing on the teaching courses belonging to the humanistic group, these findings suggest that both the adequacy of teaching material and the clearness of examination procedures exhibit a remarkable improvement when there has been the shift to distant learning.

**Table 2:** Item description

	Item wording
Item 1	Adequacy of teaching material
Item 2	Consistency of teaching with website program
Item 3	Respect for the schedule of lessons, exercises, didactic activities
Item 4	Clearness of examination procedures
Item 5	Teacher’s ability in motivating interest in the discipline
Item 6	Teacher’s ability in explaining the subject arguments
Item 7	Teacher’s availability in providing clarifications on the topics

**Table 3:** Item fit statistics at time $T_{0}$

Item	chisq	df	*p*-value	Outfit MSQ	Infit MSQ	Outfit t	Infit t
Item 1_T0	20.260	30	0.910	0.654	0.88	− 0.33	− 0.45
Item 2_T0	13.233	30	0.997	0.427	0.573	− 1.77	− 2.26
Item 3_T0	24.122	30	0.766	0.778	0.938	− 0.51	− 0.19
Item 4_T0	68.797	30	0.000	2.219	1.802	2.30	3.08
Item 5_T0	16.500	30	0.978	0.532	0.718	− 1.33	− 1.37
Item 6_T0	18.446	30	0.951	0.595	0.741	− 1.27	− 1.19
Item 7_T0	12.743	30	0.998	0.411	0.766	− 0.45	− 0.77

**Table 4:** Item fit statistics at time $T_{1}$

Item	chisq	df	*p*-value	Outfit MSQ	Infit MSQ	Outfit t	Infit t
Item 1_T1	23.097	27	0.680	0.825	1.034	0.00	0.23
Item 2_T1	28.718	27	0.375	1.026	1.028	0.19	0.20
Item 3_T1	76.595	27	0.000	2.736	1.023	1.67	0.18
Item 4_T1	27.494	27	0.437	0.982	1.145	0.09	0.74
Item 5_T1	14.254	27	0.979	0.509	0.652	− 1.34	− 1.87
Item 6_T1	11.528	27	0.996	0.412	0.537	− 1.93	− 2.41
Item 7_T1	8.777	27	1.000	0.313	0.874	0.15	-0.02

**Table 5:** Item parameter estimates at time point $T_{0}$ and $T_{1}$

Item	Difficulty parameter at T0	SE	Difficulty parameter at T1	SE
Item 1	1.642	0.45	2.184	0.471
Item 2	0.493	0.403	0.377	0.441
Item 3	− 0.793	0.415	− 1.628	0.587
Item 4	0.675	0.406	0.936	0.433
Item 5	0.493	0.403	0.936	0.433
Item 6	0.129	0.402	0.377	0.441
Item 7	− 2.639	0.557	− 3.182	0.929

**Table 6:** Trend parameter estimates

Item	$\tau _{j}$	SE	Lower CI	Upper CI	Sign.
Item 1	1.299	0.651	0.023	2.576	***
Item 2	1.609	0.632	0.37	2.849	***
Item 3	2.047	1.005	0.009	3.026	***
Item 4	1.099	0.516	0.086	2.111	***
Item 5	1.03	0.521	0.008	2.051	***
Item 6	1.253	0.567	0.142	2.364	***
Item 7	2.079	1.061	0.004	4.158	***

Sign. codes: ***0.05

## Conclusion and research limitations

Closure and distancing measures caused by the coronavirus outbreak had major impacts on teaching and learning in the university context. Firstly, face-to-face instruction has been converted to online learning. Secondly, lockdowns made households the prime learning space for students. In a nutshell, higher education systems have undergone and will undergo a digital revolution: conference call, online lectures, online exam and communications in virtual environments, will persist in the post-pandemic world. Since e-learning became the unique system for instruction, the concerns are centered on teacher’s and learner’s experience with this technology and the improvement for the future, and on examining whether this method is effective, especially when compared to face-to-face lessons. To this end, in this research, we analysed data collected through a survey administrated before and during COVID-19 pandemic to obtain accurate opinions on 41 courses taught at University of Chieti-Pescara (Italy). An IRT modelling has been adopted to study response data obtained through a set of items of a questionnaire. Specifically, we relied on IRT models with relaxed assumptions to understand if significant change occurred in the feedback on learning experience after the unplanned and rapid interruption of face-to-face education due to COVID-19 pandemic. A fundamental difference from traditional IRT calibration, that characterizes the approach followed in this study, is the estimation of the change in IRT model parameter estimates which may occur in two temporal administrations of the same questionnaire. Given the responsibilities of universities to maintain an optimal quality service, identifying which are the elements that influence students’ satisfaction and a reliable assessment of their change over time, can improve the measurement of education outcomes when higher education institutions have to face transformations, such as the digital one caused by the restrictions generated by COVID-19. The analysis carried out by means of LLRA modelling is well suited for capturing response shift effect in students assessing their learning experience in two different measurement occasions. Additionally, the adopted IRT model makes also possible to quantify the dependence of temporal changes on some covariates effect. In such way, for the problem at hand, educators will be provided with a valuable feedback for adopting strategies to ensure the excellence of the distant learning.

Overall, this study has shown that, in this first experience, students had a positive adaptation to online teaching and learning. Our survey findings have in fact demonstrated the satisfaction with the courses or lesson activities delivered in online environment. Positive feedbacks of online teaching experiences are mainly related to instructors’ availability for questions and comments and teacher’s respect or schedule of lessons, exercises, didactic activities. These results are in line with what has already highlighted the literature, from other researchers in different countries and universities. In this respect, Crews and Butterfield (2014) state that the most positive impact with online learning experiences is the class structure that supports flexibility, organization, and clear expectations. Different reasons have led us towards the research carried out in this study. Basically, our first objective is of course related to the necessity of monitoring and improving the teaching. Likewise important is the awareness that COVID-19 pandemic has drastically changed the lives of people all over the world; thus, the effectiveness of new teaching mode can be supportive to teaching and learning process post COVID-19. This survey attempted to quickly capture the changes imposed by the emergency precisely to support a community—that of students—potentially very vulnerable in the face of the emergency. Due to their social responsibility roles, universities are obliged to bridge the educational and social challenges of their surrounding societies; the educational systems have to drive the change in order to improve the community readiness for such mode of learning. We believe that in careful designing for learning in higher education post COVID-19 crisis, universities are prompted to adopt different forms of learning activities; in other terms, they should creatively merge online and face-to face teaching lessons. Throughout these concluding remarks, we have desired not only to propose a discussion of results but also to stress how this pilot study can be a listening opportunities of students, to make clear the main challenges of this unprecedented education scenario. However, this study is limited since analyzed data are referred to a single department while, for a more comprehensive analysis, it is convenient to enlarge the research to all departments. Additionally, no perspective of universities or faculty members was taken into consideration. The next and deeper research should also investigate the equitable and inclusive access of students to digital learning resources and if their social and emotional needs are being met. With reference to this latter aspect, some researchers show that under the conditions of autonomous learning, motivated students actually improved their academic performance. Future research should overcome these limitations to come up with a set of useful recommendations that would improve the distance teaching experience.

## Appendix

See Table 7.

**Table 7:** English version of the survey questions

Questions	Definitely	More no	More yes	Definitely
	No	Than yes	Than no	Yes
Item 1: Is the teaching material (indicated and available) suitable for the study of the subject?	$\Box$	$\Box$	$\Box$	$\Box$
Item 2: Has the teaching been carried out in a manner consistent with what was stated on the course website?	$\Box$	$\Box$	$\Box$	$\Box$
Item 3: Are the schedules of lessons, exercises and any other teaching activities respected?	$\Box$	$\Box$	$\Box$	$\Box$
Item 4: Have the examination procedures been clearly defined?	$\Box$	$\Box$	$\Box$	$\Box$
Item 5: Does the teacher stimulate/motivate interest in the discipline?	$\Box$	$\Box$	$\Box$	$\Box$
Item 6: Does the teacher explain the topics properly to the complexity of the subject?	$\Box$	$\Box$	$\Box$	$\Box$
Item 7: Is the teacher available during the lesson and/or at the reception time to provide clarification on the topics covered?	$\Box$	$\Box$	$\Box$	$\Box$
